# AIE-Active Photosensitizers: Manipulation of Reactive Oxygen Species Generation and Applications in Photodynamic Therapy

**DOI:** 10.3390/bios12050348

**Published:** 2022-05-18

**Authors:** Hao Yu, Binjie Chen, Huiming Huang, Zhentao He, Jiangman Sun, Guan Wang, Xinggui Gu, Ben Zhong Tang

**Affiliations:** 1 Beijing Advanced Innovation Center for Soft Matter Science and Engineering, College of Materials Science and Engineering, State Key Laboratory of Chemical Resource Engineering, Beijing University of Chemical Technology, Beijing 100029, China; 2019201328@buct.edu.cn (H.Y.); 2021200565@buct.edu.cn (B.C.); 2019210862@buct.edu.cn (H.H.); 2020210841@buct.edu.cn (Z.H.); sunjiangman@buct.edu.cn (J.S.); 2 Beijing National Laboratory for Molecular Sciences, Beijing 100190, China; 3 Shenzhen Institute of Aggregate Science and Technology, School of Science and Engineering, The Chinese University of Hong Kong (Shenzhen), Shenzhen 518172, China; tangbenz@cuhk.edu.cn

**Keywords:** aggregation-induced emission, photosensitizer, reactive oxygen species, photodynamic therapy, aggregation microenvironment

## Abstract

Photodynamic therapy (PDT) is a non-invasive approach for tumor elimination that is attracting more and more attention due to the advantages of minimal side effects and high precision. In typical PDT, reactive oxygen species (ROS) generated from photosensitizers play the pivotal role, determining the efficiency of PDT. However, applications of traditional PDT were usually limited by the aggregation-caused quenching (ACQ) effect of the photosensitizers employed. Fortunately, photosensitizers with aggregation-induced emission (AIE-active photosensitizers) have been developed with biocompatibility, effective ROS generation, and superior absorption, bringing about great interest for applications in oncotherapy. In this review, we review the development of AIE-active photosensitizers and describe molecule and aggregation strategies for manipulating photosensitization. For the molecule strategy, we describe the approaches utilized for tuning ROS generation by attaching heavy atoms, constructing a donor-acceptor effect, introducing ionization, and modifying with activatable moieties. The aggregation strategy to boost ROS generation is reviewed for the first time, including consideration of the aggregation of photosensitizers, polymerization, and aggregation microenvironment manipulation. Moreover, based on AIE-active photosensitizers, the cutting-edge applications of PDT with NIR irradiated therapy, activatable therapy, hypoxic therapy, and synergistic treatment are also outlined.

## 1. Introduction

Light has been used in the treatment of diseases for thousands of years. In ancient Egypt, *Ammi majus* Linnaeus was used to treat vitiligo, followed by exposing the patients to sunlight, and extracts from certain plants that contained photosensitizers were expanded to deal with other skin diseases. In the early 20th century, von Tappeiner discovered that photosensitizers could be used with light to kill cells, and defined this process as photodynamic therapy (PDT). Thereafter, the oxygen-dependence of PDT was revealed and the foundational principles of PDT were first described [1,2]. PDT became a special therapeutic strategy in treating patients, and the development of PDT as a non-invasive therapy flourished in the following decades. 

PDT is generally implemented with the basic components of photosensitizers, light, and oxygen. Each component is basically non-toxic to the human body, and PDT can precisely control the killing of cancer cells and microorganisms by manipulating the wavelength, intensity, and irradiation range of light to reduce side effects in the treatment process, thereby boosting the development of antitumor treatments [3,4,5] and antibacterial applications [6,7,8].

The key factors for effective PDT applications are the reactive oxygen species (ROS) that originate from photosensitizers upon light irradiation. This process works by inducing tumor cell death by damaging the organelle and by suppressing cell proliferation by blocking the signaling pathway and inhibiting the cell cycle. Cell death caused by ROS has been well studied. As ROS are generated in cell, they can react with cellular components such as lipids, proteins, nucleic acids, and carbohydrates [9], leading to metabolic and cellular disturbances to achieve antitumor effects. 

Accordingly, three main models of cell death caused by ROS in PDT, involving necrosis, apoptosis, and autophagy, are proposed [10,11]. Necrosis is a kind of passive cell death caused by ROS, and generally occurs as lipids and proteins in cellar membranes are subjected to damage by ROS, leading to the destruction of membrane integrity and ion homeostasis for the necrosis of cell. Apoptosis of cells in PDT is a programmed pathway triggered by initial damage to the organelles by ROS. It generally occurs as the signaling pathways from a cell’s surface or from a site of cell damage are converged into a small number of central pathways, resulting in the final “execution” of the whole cell, followed by morphological changes in the cell [12]. Autophagy is a catabolic process that plays a pivotal role in renewing damaged organelles in cytoplasm, a mechanism that is considered important for cytoprotection. However, autophagy can also be stimulated by ROS, depending on the type of ROS and the degree of oxidative injury; alternatively, autophagy may occur abnormally by the destruction of organelles by ROS, eventually inducing cell death [11]. 

**Figure 1:**
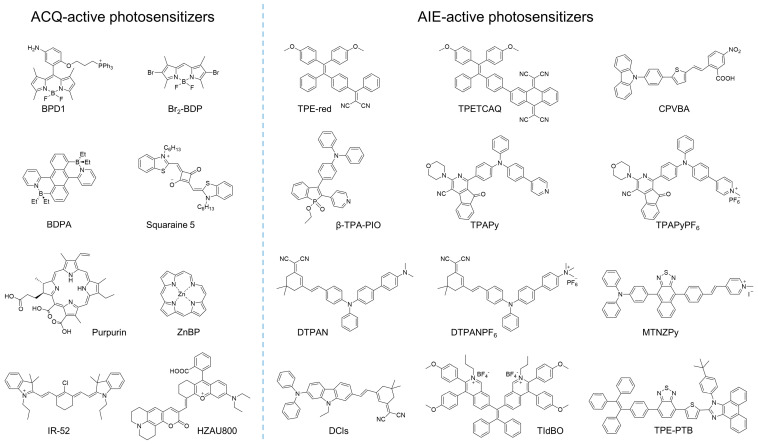
Examples of molecular structures of ACQ or AIE-active photosensitizers.

Generally, the response of cells to damage depends on several factors, such as the photosensitizer selected, the light dose applied, and the cellular metabolic state. The photosensitizer that is employed plays a critical role in the entire PDT process. Previously, many photosensitizers that involved inorganic and organic materials were developed and implemented for PDT. However, for purposes of biocompatibility and tunable photosensitization, organic photosensitizers exhibit superiority over other photosensitizers. Some examples of organic photosensitizers are provided in Figure 1. 

**Figure 2:**
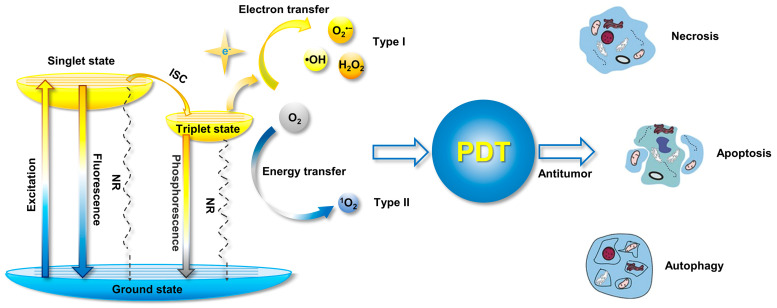
Mechanism of ROS generation form photosensitizer illustrated by Jablonski diagram, and the processes for PDT-induced cell death (NR: nonradiative pathway). Modified with the permission from the authors of [3]. Copyright 2020, the Author(s). Published by Elsevier.

With the development of organic photosensitizers in recent decades, the photosensitization mechanism has been well-studied. The general photosensitization mechanism a photosensitizer is illustrated in the Jablonski diagram, as shown in Figure 2 [13]. Upon absorbing a photon, the photosensitizer is excited into a higher-energy state from the singlet ground state (S_0_), depending on the excitation wavelength, and it relaxes to the lowest excited single state (S_1_) for the subsequent photophysical processes, according to Kasha’s rule [14]. Although the excited molecule always undergoes an S_1_ → S_0_ transition by releasing energy through a nonradiative pathway to yield heat or via radiative decay to produce fluorescence, it could also be transferred into the lowest excited triplet state (T_1_) via S_1_ → T_1_ intersystem crossing (ISC) processes, if the energy difference between S_1_ and T_1_ (Δ*E*_ST_) is small enough or if their spin orbit coupling (SOC) effect is strong. Compared with the S_1_ state, the high-energy T_1_ exhibits a longer lifetime due to the prevention of spin in T_1_ → S_0_, resulting in phosphorescence emission and possible ROS production. 

According to the pathway of ROS generation, the obtained ROS could be classified in one of two types, type I and type II. Generally, type II ROS of singlet oxygen (^1^O_2_) could be harvested from the excited triplet state via energy transfer, covering the most commonly used organic photosensitizers, while type I ROS, including superoxide anion (O_2_^•−^), hydroxyl radical (•OH), and hydrogen peroxide (H_2_O_2_) etc., are commonly obtained via a one-electron oxidation–reduction reaction with a neighboring oxygen molecule through electron transfer from an excited triplet state, which is more efficient for oxygen utilization and is usually considered to solve anoxia in the tumor microenvironment during PDT.

Although PDT has been implemented in the treatment of disease for a long time, the first organic photosensitizers were developed in the 1970s, and were considered to be the typical first-generation photosensitizers. They included a hematoporphyrin derivative (HpD) (containing a proprietary mix of porphyrin monomers, dimers, and oligomers) with porfimer sodium (the active material in HpD). Although they were the earliest and most useful photosensitizers in clinical trials, these photosensitizers suffered from relatively weak absorption of light, especially infrared or near-infrared (NIR) absorption. Such defects, as well as the unknown structures of the first-generation photosensitizers, hindered further applications, and encouraged the development of second-generation photosensitizers. Porphyrinoid compounds comprising porphyrin or porphyrin-based macrocyclic analogues (such aschlorins and bacteriochlorins) and nonporphyrinoid compounds (anthraquinones, phenothiazines, and curcuminoids) with identified chemical structures were the second-generation photosensitizers [15]. These photosensitizers usually exhibited expanded absorption with wavelengths longer than 630 nm, as well as high extinction coefficients. Compared to the first-generation photosensitizers, the second-generation photosensitizers presented higher quantum yields of ^1^O_2_, a higher tumor-to-normal tissue ratio, and, accordingly, a better antitumor effect. However, due to their hydrophobicity and lack of targeting, the applications of second-generation photosensitizers were also greatly limited. Many researchers in the field focused on developing third-generation photosensitizers with infrared absorption, better tumor specificity, and higher ROS generation. 

In order to improve the efficiency of PDT, it is important to design photosensitizers that enhance ROS production. According to the mechanism of photosensitization, ROS are commonly generated from the excited triplet state via energy or electron transfer to surrounding oxygen molecules. Many strategies have been proposed to boost ROS generation, in which aggregation is one of the strategies for developing the photosensitizers required by promoting intersystem crossing (ISC) [16]. With appropriate aggregation, the enhanced photosensitization capability of various traditional photosensitizers, such as the photosensitization of pentamethine indocyanine [17], phthalocyanine [1,18], and porphyrin [19], has been demonstrated. However, due to the hydrophobicity of traditional photosensitizers, they aggregate in physiological circumstances, with aggregation-caused quenching (ACQ) effects that make it difficult to enhance ROS generation via aggregation because of the strong π-π interaction in aggregates [5]. Although scientists have developed a number of strategies to balance the notorious ACQ effects and aggregation-enhanced ROS generation, such as molecular modification [20] and polymer isolation [21], most of these strategies relied heavily on complicated chemical syntheses or low concentrations of photosensitizers in the photodynamic agents. 

**Figure 3:**
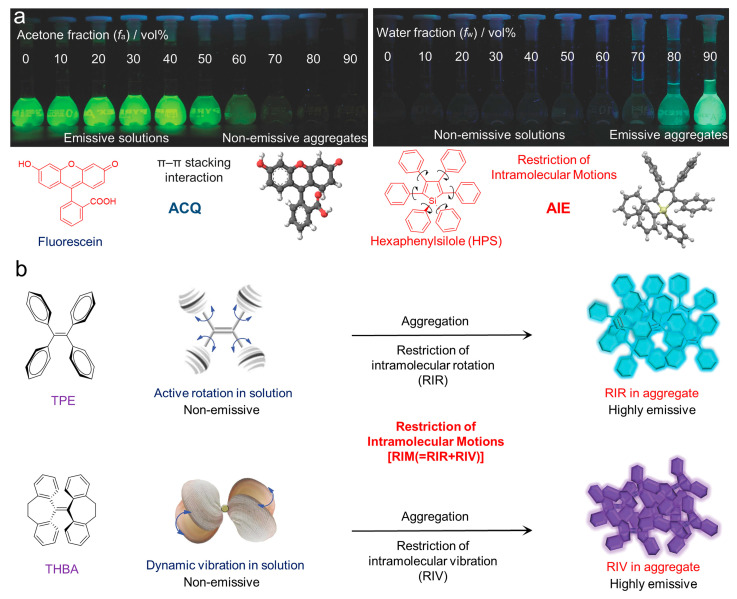
(**a**) Fluorescent photographs of the ACQ and AIE molecules as they aggregated in solution; (**b**) schematic illustration of the mechanisms of the ACQ and AIE phenomenon. Reprinted with the copyright from [24]. Copyright 2014, Wiley-VCH GmbH.

Fortunately, the concept of aggregate-induced emission (AIE) was proposed in 2001 [22,23,24,25]. Luminogens with aggregation-induced emission characteristics (AIEgens) commonly emitted weakly in solution, while they would exhibit intense fluorescence as they aggregated (Figure 3a), overcoming the obstacle of ACQ as the dyes aggregated and providing new opportunities for constructing functional luminogens. To achieve insights into AIE, much effort was devoted by researchers, and the primary mechanism for interpreting the AIE phenomenon was widely regarded as the restriction of intramolecular motions (RIM), including the restriction of intramolecular rotations (RIR) and the restriction of intramolecular vibrations (RIV), as shown in Figure 3b. As the photosensitizers were designed with AIE characteristics, they could be utilized in a high concentration for pursuing effective PDT without the disadvantages of the ACQ effects. Therefore, photosensitizers with AIE characteristics have been well-studied, and numerous of AIE-active photosensitizers with effective ROS generation and infrared absorption have been developed, boosting the development of PDT. A number of reviews have been published to summarize the development of AIE-active photosensitizers and the subsequent PDT, which made it convenient for us in understanding the progress of related fields [26,27,28,29,30,31,32,33,34]. However, those reviews concentrated mostly on the applications of PDT, with less focus on the design of AIE-active photosensitizers and manipulation of ROS generation. Hence, in this review, we focus on the AIE-active photosensitizer by summarizing the strategies for designing and manipulating ROS generation and related advanced therapy strategies adopted for PDT in recent years.

## 2. Manipulated Photosensitization of AIE-Active Photosensitizers

### 2.1. Manipulation Based on Molecular Design

#### 2.1.1. Attaching Heavy Atoms to the Molecular Skeleton

According to the mechanism of ROS production, ISC processes from excited singlet states to excited triplet states dominate ROS generation. Previous literature [35] indicated that ISC rate constants could be estimated from Equation (1), where H_SO_ is the Hamiltonian for the spin–orbit perturbations (SOP) and Δ*E*_ST_ is the energy gap between the S_1_ and T_1_ states.

(1) $$K_{\mathrm{ISC}}\propto \frac{\langle T_{1}\left|H_{\mathrm{SO}}\right|S_{1}\rangle {}^{2}}{\Delta E_{\mathrm{ST}}}$$

**Figure 4:**
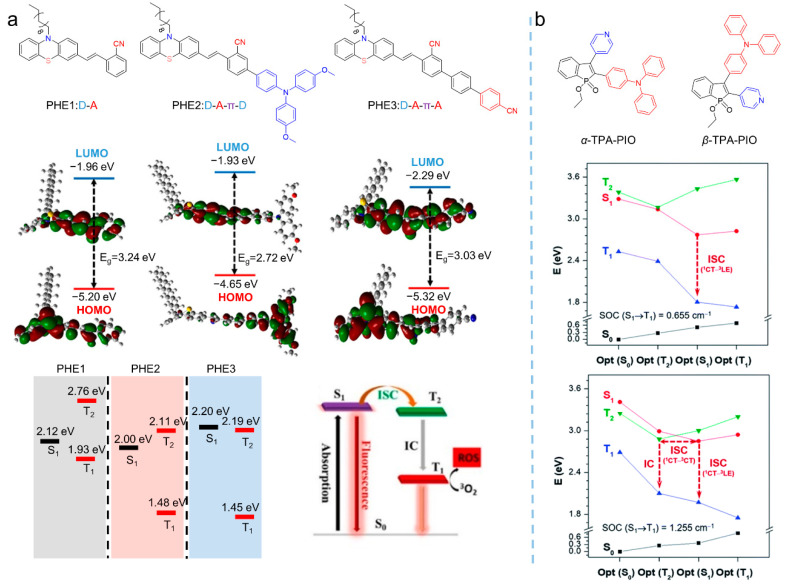
(**a**) Chemical structures, the electron distribution on the frontier orbitals, and the relied mechanism for PHE1-3 to produce type I ROS. Reprinted with the copyright from. [37]. Copyright 2021, Elsevier. (**b**) Chemical structures and calculated energy diagram analysis of *α*-TPA-PIO and *β*-TPA-PIO with labels of SOC values. Reprinted with the copyright from [38]. Copyright 2020 by the author(s). Published by the Royal Society of Chemistry.

Note that attaching heavy atoms to chromophores favors H_SO_ by enhancing SOP, leading to higher ISC rates. A heavy atom effect is the commonly used strategy in a wide range of molecular structures to yield an effective photosensitizer by prompting the ISC processes via an SOC process between singlet and triplet [36]. Various AIE-active photosensitizers have been developed with efficient photosensitization by introducing heavy atoms into chemical structures. For example, Xu et al. [37] synthesized a series of photosensitizers—PHE1 (*λ*_abs_ = 420 nm, *λ*_em_ = 581 nm), PHE2 (*λ*_abs_ = 414 nm, *λ*_em_ = 578 nm), and PHE3 (*λ*_abs_ = 429 nm, *λ*_em_ = 608 nm)—by taking advantage of the heavy atom effect that originated from sulfur and nitrogen atoms, as shown in Figure 4a. The mechanism in ROS generation could be assigned to the enhancement of the ICT by the heavy-atom, which facilitated ISC processes to generate triplet excitons and the generation of O_2_^•−^. Similarly, aided by the unique electronic structure of phosphindole oxide (PIO) and the heavy atom effect of the phosphorus atom in PIO, Tang et al. [38] introduced triphenylamine and pyridine groups in the molecular structure of PIO and synthesized the two isomers, *α*-TPA-PIO (*λ*_abs_ = 411 nm, *λ*_em_ = 563 nm) and *β*-TPA-PIO (*λ*_abs_ = 393 nm, *λ*_em_ = 560 nm), as shown in Figure 4b. According to their results, *β*-TPA-PIO exhibited an efficient generation of type I ROS, both in solution and in cells. Furthermore, the theoretical calculation studies revealed that the efficient intersystem crossing and electrophilic ability of *β*-TPA-PIO provided it with type I ROS generation ability. However, the introduction of heavy atoms was always followed by the unexpected cytotoxicity, which limited the further development of this strategy; many alternative approaches were proposed [39].

#### 2.1.2. Constructing Donor-Acceptor Effect in Molecular Structures

**Figure 5:**
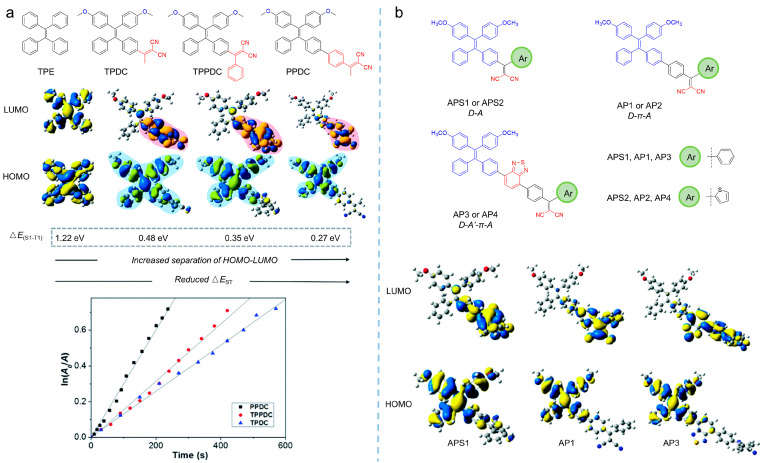
Donor-acceptor molecular engineering strategies to enhance ROS. (**a**) Molecular structure, calculated HOMO-LUMO distributions, and ROS generation of TPDC, TPPDC, and PPDC. Reprinted with the permission the authors of [41]. Copyright 2015, by the author(s). Published by the Royal Society of Chemistry. (**b**) Molecular structure with extended π-spacer between donor and acceptor and calculated HOMO-LUMO distributions of the TPE-based photosensitizers. Reprinted with the permission of the authors of [42]. Copyright 2017, Royal Society of Chemistry.

From Equation (1), it can be found that the rate of ISC is inversely proportional to Δ*E*_ST_, which implied that ISC processes could be promoted by reducing Δ*E*_ST_. The smaller the Δ*E*_ST_, the higher the ISC rate. Further, according to photophysical principles, the effective reduction of Δ*E*_ST_ relies on the separation of the highest energy occupied molecular orbital (HOMO) and the lowest energy unoccupied molecular orbital (LUMO) distribution [40]. On that basis, numerous approaches to the promotion the photosensitization by reducing Δ*E*_ST_, such as constructing donor-acceptor moieties into the molecular structure and extending the π-spacers between donor and acceptor, have been developed. Based on the typical AIE molecule of tetraphenylethylene (TPE), Liu et al. [41] synthesized a series of photosensitizers—TPDC (*λ*_ex_ = 400 nm, *λ*_em_ = 602 nm), TPPDC (*λ*_ex_ = 390 nm, *λ*_em_ = 632 nm), and PPDC (*λ*_ex_ = 420 nm, *λ*_em_ = 595 nm)—through molecular engineering strategies by inducing donor-acceptor moieties into the molecular structure, as shown in Figure 5a. The results showed that the Δ*E*_ST_ of the photosensitizers was reduced from 1.22 eV to 0.27 eV, with the donor-acceptor interactions enhanced, and a 3.2-fold improvement in ^1^O_2_ yield was achieved, indicating the potential to enhance ROS generation by introducing the donor and the acceptor into molecular photosensitization. 

Extending the π-spacer between the donor and the acceptor is another strategy to reduce the Δ*E*_ST_ of photosensitizers by separating the HOMO and LUMO. In Figure 5b, three TPE-based photosensitizers are shown with the difference of the π-spacer between the acceptor and the donor [42]. By enlarging the π-spacer, HOMO and LUMO becoming separated, leading to the Δ*E*_ST_ being varied from 0.33 to 0.30 eV, and the triplet state increased beyond 0.98 eV of ^1^O_2_, facilitating the production of ^1^O_2_. Further, the addition of the π-spacer would extend the red-shift of the synthesized photosensitizers on the absorption tail, from 530 nm to 600 nm, benefiting their applications in PDT. 

#### 2.1.3. Manipulating ROS Generation upon Ionization

**Figure 6:**
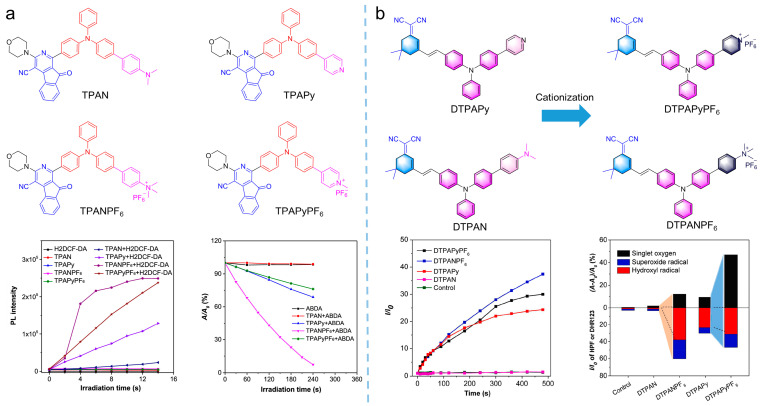
Cationic molecular engineering strategy to enhance ROS. (**a**) Molecular structure and ROS generation of TPAN, TPAPy, TPANPF_6_ and TPAPyPF_6_ indicated by H2DCF-DA and ABDA. Reprinted with the permission of the authors of [43]. Copyright 2019, American Chemical Society. (**b**) Molecular structure, ROS generation of DTPAPy, DTPAN, DTPAPyPF_6_, and DTPANPF_6_ indicated by HPF or DHR123, and summary of different ROS generation of photosensitizers. Reprinted with the permission of the authors of [44]. Copyright 2022, Elsevier.

Currently, the introduction of donor-acceptor moieties and heavy atoms into photosensitizers is widely used to enhance ROS by reducing the Δ*E*_ST_ and promoting ISC processes. However, little attention has been paid to the typological alternation of ROS. Due to hypoxic microenvironment, type I ROS of O_2_^•−^, •OH, and H_2_O_2_, are more adaptive to tumor treatment with oxygen independence, generating widespread attention. According to the mechanism of ROS generation, type I ROS are produced by a charge transfer process. Therefore, improving the electron affinity of photosensitizers and promoting the electron capture of molecules are of significance; introducing ionization to photosensitizers would facilitate the generation of type I ROS. Tang et al. [43] proposed an effective strategy for improving photosensitizer performance through cationization. As shown in Figure 6a, a series of photosensitizers of TPAN, TPAPy, TPANPF_6_, and TPAPyPF_6_ were synthesized by using the morpholine-modified nitrofluorene acceptor and the triphenylamine donor. By altering the different substituents, including dimethylaniline, pyridine, trimethylphenylammonium hexafluorophosphate, and 1-methylpyridin-1-ium hexafluorophosphate, the ionization effect on ROS generation was investigated. TPAN (*λ*_abs_ = 417 nm, *λ*_em_ = 601 nm) was incapable of producing ROS, while TPAPy (*λ*_abs_ = 409 nm, *λ*_em_ = 593 nm) could only produce ^1^O_2_. By introducing the ionization effect, ROS generation was enhanced by TPANPF_6_ (*λ*_abs_ = 408 nm, *λ*_em_ = 595 nm) and TPAPyPF_6_ (*λ*_abs_ = 437 nm, *λ*_em_ = 578 nm) molecules, which were modified by a positive charge, with an excellent O_2_^•−^. This work demonstrated that the introduction of cations could separate molecular charges and capture electrons more easily, benefitting the generation of type I ROS. Furthermore, Tang et al. [44] synthesized two molecules (DTPAPy (*λ*_abs_ = 495 nm, *λ*_em_ = 675 nm) and DTPAN) and ionized them to produce DTPANPF_6_ (*λ*_abs_ = 498 nm, *λ*_em_ = 675 nm) and DTPAPyPF_6_ (*λ*_abs_ = 480 nm, *λ*_em_ = 675 nm), as shown in Figure 6b, which exhibited excellent type I and type II ROS generation performance.

**Figure 7:**
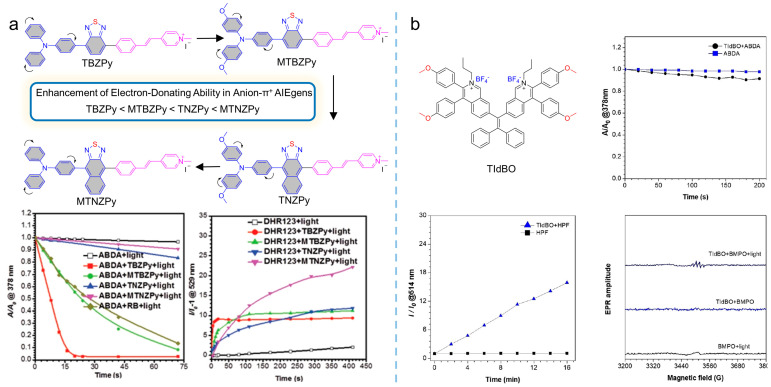
Anionization strategy to enhance ROS generation. (**a**) Molecular structure and ROS generation of TBZPy, MTBZPy, TNZPy, and MTNZPy indicated by ABDA and DHR123. Reprinted with the permission of the authors of [45]. Copyright 2020, Wiley-VCH GmbH. (**b**) Molecular structure, ROS generation of TIdBO indicated by ABDA, HPF, and EPR spectra of the TIdBO. Reprinted with the permission of the authors of [46]. Copyright 2021, American Chemical Society.

Anionization could also be employed to tune the generation of type I ROS, and the mechanism was always assigned to efficient ISC to ensure sufficient triplet energy generation and a rich electron environment to supply electrons to the excited photosensitizers. Tang et al. [45] synthesized four molecules of TBZPy (*λ*_abs_ = 447 nm, *λ*_em_ = 627 nm), MTBZPy (*λ*_abs_ = 476 nm, *λ*_em_ = 653 nm), MTNZPy (*λ*_abs_ = 528 nm, *λ*_em_ = 686 nm), and TNZPy (*λ*_abs_ = 511 nm, *λ*_em_ = 662 nm) with iodide ion as the coordination system, as shown in Figure 7a. The four molecules exhibited a classic donor-acceptor structure, where the donors were composed of benzo-2,1,3-thiadiozole (BZ)/naphtho [2,3-c][1,2,5]thiadiazole (NZ) groups modified with triphenylamine and its methoxy-substituted derivatives, and the acceptors were comprised of styrenylpyridine. TBZpy could only produce ^1^O_2_, due to the weak intramolecular charge transfer (ICT) effect, while the enhancement of electron-donor ability enabled the other molecules to produce other types of ROS, which demonstrated that iodide ions and highly reductive donors provided the electron-rich aggregation microenvironment for excited photosensitizers, promoting the production of type I ROS. Similarly, as shown in Figure 7b, Tang et al. [46] synthesized the photosensitizer of TIdBO (*λ*_abs_ = 372 nm, *λ*_em_ = 547 nm), an isoquinoline organic salt derivative with excellent photosensitization performance. Owing to the strong ICT that originated from the anion−π^+^ effect and the cyclization reaction upon irradiation, oxygen molecules in the surrounding environment participated in this electron transfer process and interacted with intermediate active radicals, generating type I ROS. ROS measurement revealed that the anionic TIdBO exhibited significant ROS production, with the intense generation of •OH and the weak generation of ^1^O_2_.

#### 2.1.4. Switching the ROS Generation by Activation of Photosensitization

Although photosensitizers with AIE characteristics have been developed rapidly, they have not yet completely overcome the shortcomings of traditional photosensitizers. As photosensitizers were injected into circulatory systems, they remained in an “always-on” state to activate and kill cancer and normal cells indistinguishably under light irradiation, and these side effects greatly weakened the treatment of the diseases. Therefore, it was necessary to develop a new type of activatable photosensitizer for the special circumstances [47,48].

**Figure 8:**
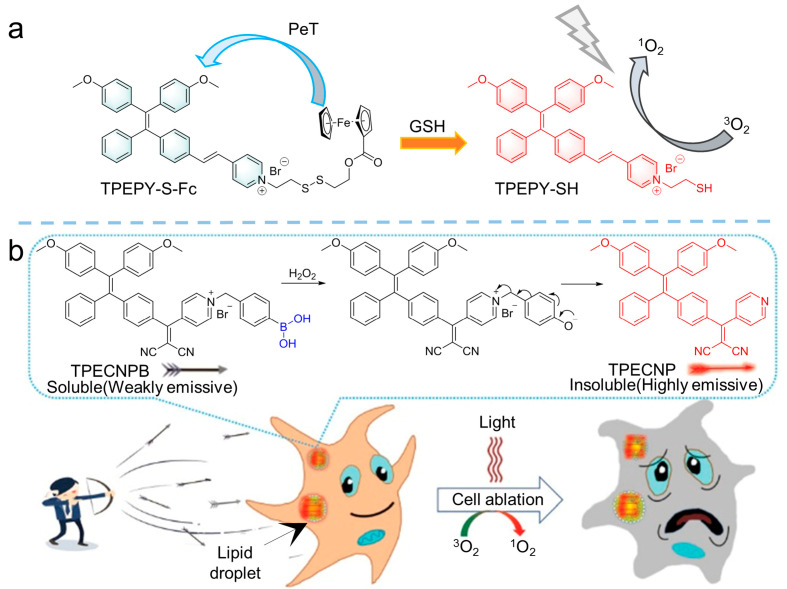
Activatable photosensitizer strategy to manipulate ROS generation by GSH and H_2_O_2_. (**a**) Molecular structure of TPEPY-S-Fc and the proposed mechanism of GSH-activated PDT. Reprinted with the permission of the authors of [57]. Copyright 2020, Royal Society of Chemistry. (**b**) Molecular structure of TPECNPB and the schematic illustration of H_2_O_2_ activation of PDT. Reprinted with the permission of the authors of [61]. Copyright 2020, Wiley-VCH GmbH.

Unlike normal cells, specific tumor microenvironments, such as pH [49,50], specific enzymes [51], and high concentrations of glutathione (GSH) [52], have received widespread attention from researchers in designing activatable photosensitizers based on tumor microenvironment activation strategies. Previous studies showed that GSH could react with a disulfide (S-S) bond [53], providing the “switch” for an activatable photosensitizer. Meanwhile, due to the interaction of GSH and intracellular ROS, the consumption of GSH leads to an improvement of intracellular ROS and PDT efficiency [54,55,56]. Therefore, employing GSH to activate photosensitizers is an appropriate strategy. As shown in Figure 8a, Kim et al. [57] reported that a TPE derivative comprising an AIE molecule (TPEPY-S-Fc) was developed as a GSH-activated photosensitizer for PDT. As shown in Figure 8a, TPEPY-S-Fc was synthesized by covalently conjoining a TPE derivative with ferrocene diethylene and vinylpyridine by disulfide bonds. As a well-known quenching agent, ferrocene could quench the fluorescence of organic fluorophores through a photo-induced electron transfer (PET) process. Therefore, the fluorescence and the ROS production of TPEPY-S-Fc was blocked by ferrocene. As GSH added, the S-S bond was split to produce an activatable photosensitizer of TPEPY-SH (*λ*_abs_ = 430 nm, *λ*_em_ = 620 nm) with the fluorescence enhanced, which could be used for effective PDT. Based on this strategy, various activatable photosensitizers have been developed [58,59].

Similarly, excessive H_2_O_2_ in tumors’ microenvironment could also be employed as a factor to active photosensitization [60]. For example, as shown in Figure 8b, Wang et al. [61] synthesized the H_2_O_2_ activatable amphiphilic photosensitizer of TPECNPB, comprising the positively charged pyridine pendant that could target the negatively charged lipid droplets by electrostatic interactions. However, in this molecule, boronate attached to the pyridine chain would be split by H_2_O_2_ and subsequently release the hydrophobic AIE-active photosensitizer of TPECNP (*λ*_ex_ = 450 nm, *λ*_em_ = 625 nm). After that, ROS generation was triggered and emitted red emission, leading to fluorescence-guided and activatable PDT.

**Figure 9:**
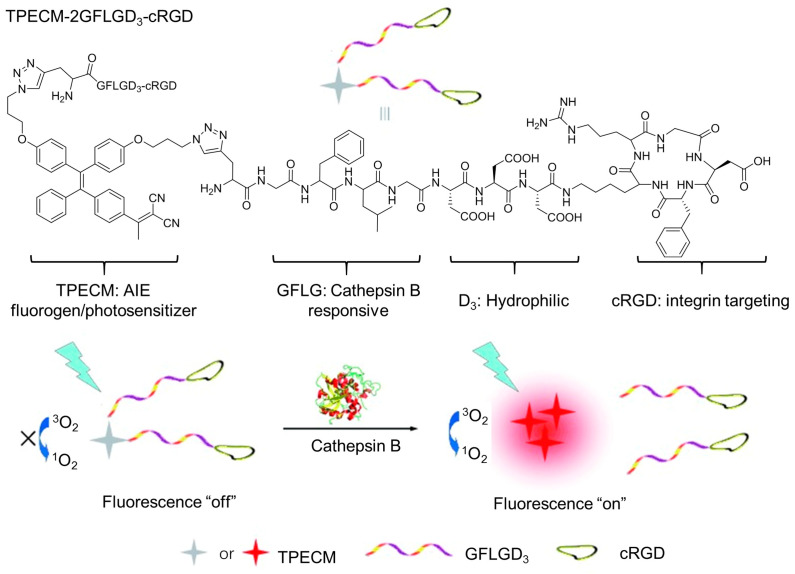
Activatable photosensitizer strategy to manipulate ROS by cathepsin B and pH. Structure of the functionalize TPE derivative TPECM and the bioprobe TPECM-2GFLGD3-cRGD, and the schematic illustration of probe activation by cathepsin B. Reprinted with the permission of the authors of [63]. Copyright 2015, Wiley-VCH GmbH.

Cathepsin B is a lysosomal protease that is always overexpressed in a variety of tumors [62]. Generally, cathepsin B can cleave polypeptide with Gly-Phe-Leu-Gly (GFLG) sequences, providing the possibility of designing photosensitizers activated by cathepsin B. Liu et al. [63] developed an activatable photosensitizer of TPECM-2GFLGD_3_-cRGD initiated by cathepsin B (Figure 9). The photosensitizer was composed by the following four parts: (a) the AIE-active TPECM as a photosensitizer; (b) GFLG peptides as trigger points; (c) hydrophilic units used for increasing hydrophilicity; and (d) cRGD as targeting units. The AIE characteristic of the photosensitizer, TPECM-2GFLGD_3_-cRGD, exhibited almost no fluorescence in aqueous media and low ROS generation. After it was taken by cancer cells, intercellular cathepsin B lysed the GFLG sequence, resulting in the release and aggregation of TPECM, which enhanced its fluorescence emission and simultaneously triggered ROS production.

**Figure 10:**
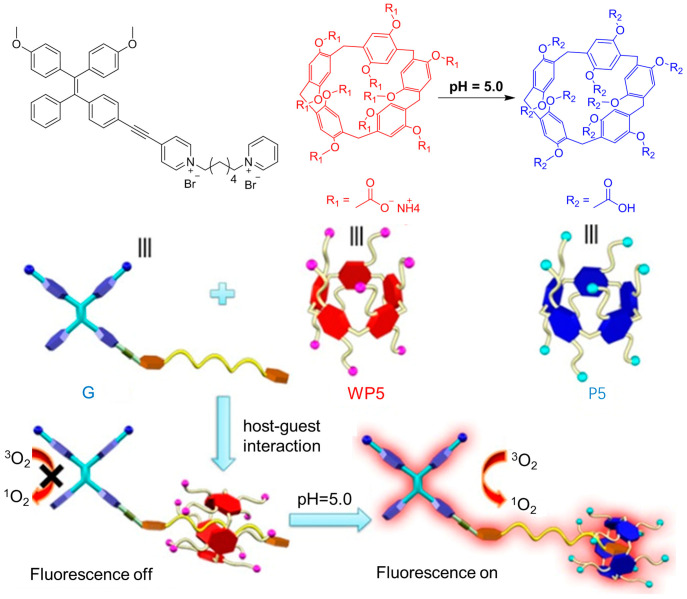
Molecular structure and schematic illustration of the molecule of WP5, P5, and G, and the proposed mechanism of pH activation of PDT. Reprinted with the permission of the authors of [66]. Copyright 2020, Wiley-VCH GmbH.

The pH response of AIE-active molecules is an alternative strategy for constructing an activatable photosensitizer [64,65]. For example, Huang et al. [66] developed a new method to achieve an activatable photosensitizer through pH-responsive supramolecular host-guest interactions. As shown in Figure 10, molecule G consists of three parts: an AIE unit of TPE, an electron-deficient pyridine unit, and a hexyl chain with another pyridine unit at the end. In molecule G, TPE acts as an electron donor, and the pyridine was taken as the electron acceptor, which separated HOMO and LUMO to promote the ICT from the S_1_ to T_1_ states for ROS generation. However, after modifying the G molecule with anionic water-soluble column aromatic hydrocarbons (WP5), a supramolecular construction of host-guest interactions between molecule G and WP5 was established, and the PET process between molecule G and WP5 inhibited the production of fluorescence and ROS. Due to the hydrophilic-hydrophobic transition of carboxyl groups, WP5, as well as the supramolecular construction, exhibited pH sensitivity at pH 5.0. Hence, by changing the environmental pH, the PET interaction between G molecules and WP5 molecules could be regulated, realizing activatable ROS production.

### 2.2. Manipulation Based on Aggregation

Aggregation of elements usually leads to a unique aggregation microenvironment within the aggregates, involving intermolecular interactions, a confined space, and so on, which greatly promote the photosensitization properties of the aggregates [67]. The strategy of aggregation has been employed to enhance ROS production for traditional photosensitizers. While such a strategy would be more appropriate for AIE-active photosensitizers, there are two main reasons for this enhancement originating from aggregation: (1) the energy transfer from S_1_ to T_1_ would be facilitated, as the energy dissipation through nonradiative channels are inhibited as the photosensitizers aggregated; and (2) aggregation would effectively enhance ISC processes [16,41,68].

#### 2.2.1. Molecule Aggregation

**Figure 11:**
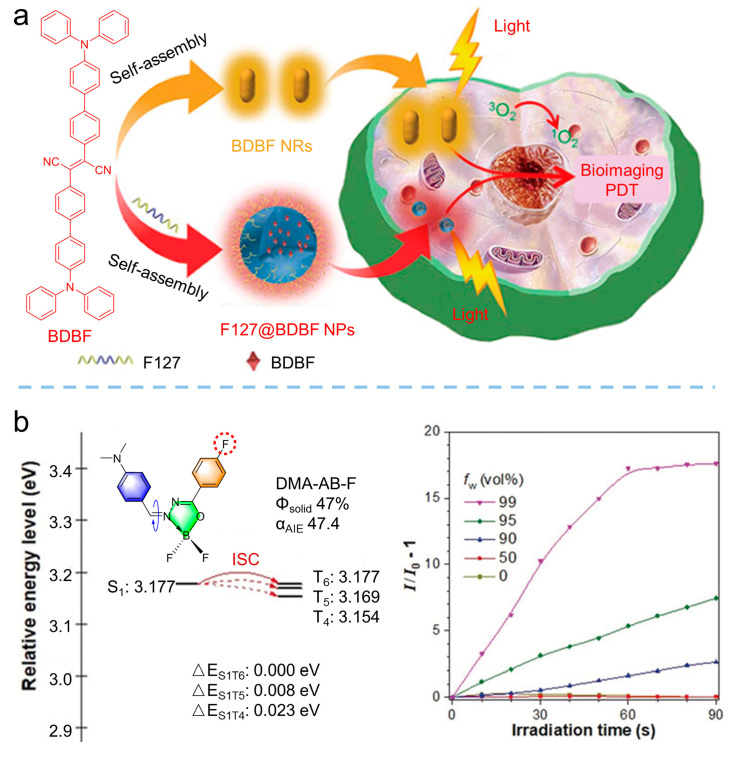
Molecule aggregation strategy to enhance ROS. (**a**) Molecular structure of BDBF and schematic illustration of BDBF NRs and F127@BDBF NPs for image-guided PDT. Reprinted with the permission of the authors of [69]. Copyright 2020, Springer Nature. (**b**) Molecular structure of DMA-AB-F and G and changes in ROS production with different degrees of aggregation. Reprinted with the permission of the authors of [70]. Copyright 2020, Wiley-VCH GmbH.

AIE-active photosensitizers overcome the deficiency of traditional photosensitizers, making it easier to implement aggregation strategies to tune the photosensitizers. For example, Tian et al. [69] synthesized the AIE-active photosensitizer of 2,3-bis(4ʹ-(diphenylamino)-[1,1ʹ-biphenyl]-4-yl) fumaronitrile (BDBF, *λ*_em_ = 585 nm), as shown in Figure 11a; they found that the aggregation of BDBF in either self-assembly or in an F127 matrix facilitated the generation of ^1^O_2_. However, they did not provide a clear mechanism for this effect. Further, Li et al. [70] synthesized the planar AIEgens of DMA-AB-F (*λ*_em_ = 467 nm), as shown in Figure 11b. By analyzing the ROS production and energy level, they demonstrated that aggregation could enhance photosensitization by the suppressed nonradiative processes and the reduced energy barrier of Δ*E*_ST_.

#### 2.2.2. Polymerization

**Figure 12:**
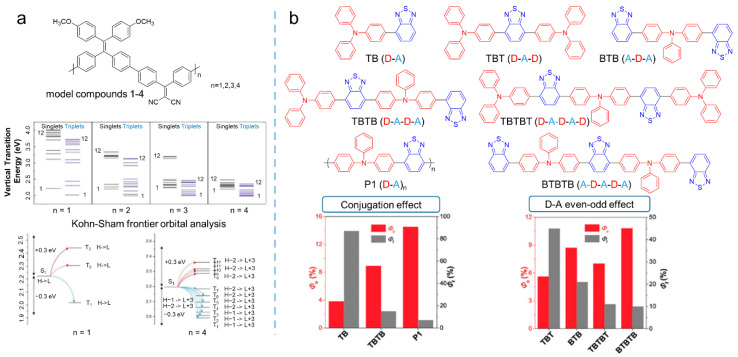
Polymerization strategy to enhance ROS generation. (**a**) Molecular structure of the model compounds, calculated energy levels, and possible ISC channels of different model compounds. Reprinted with the permission of the authors of [71]. Copyright 2018, Elsevier. (**b**) Molecular structure, ^1^O_2_ quantum yield, and fluorescence quantum yield of TB, TBTB, P1, TBT, BTB, TBTBT, and BTBTB. Reprinted with the permission of the authors of [72]. Copyright 2018, Wiley-VCH GmbH.

To some extent, polymerization could be considered as a well-organized aggregation of photosensitizers, providing a facile strategy for tailoring the ideal photosensitizer. For example, Liu et al. [71] proposed a new strategy of “polymerization-enhanced photosensitization”, with about four times greater ^1^O_2_ production than that of the small-molecule analogs as the degree of the polymer reached four units. The mechanism for the enhancement was supported by time-dependent density functional theory (TD-DFT) calculations. As shown in Figure 12a, the theoretical investigations clearly revealed that the difference between the energy levels of the upper excited states (S_n_ and T_n_) and the lowest excited states (S_1_ and T_1_) was decreased as the repeated conjugated units of the conjugated polymer increased. As a consequence, ISC processes were promoted as the energy levels grew close, which was confirmed by Kohn-Sham frontier orbital analysis. Although Liu demonstrated the feasibility of constructing polymerized photosensitizers by this proposed strategy, they ignored the influence caused by the acceptor-donor effect. Hence, Tang et al. [72] further investigated the acceptor-donor effect in polymer chains for photosensitization. In their study, triphenylamine was taken as the donor, while benzo-thiadiazole was selected as the acceptor. By the Suzuki reaction, a series of photosensitizers with the acceptor-donor structure were synthesized, and the ROS quantum yield was measured via a chemical method with Rose bengal (RB) as the standard photosensitizer. As shown in Figure 12b, an obvious enhancement in ROS quantum yield could be found as the numbers of acceptor exceeded that of donors, demonstrating the special even–odd effect in polymerization-enhanced photosensitization.

#### 2.2.3. Aggregation Microenvironment

**Figure 13:**
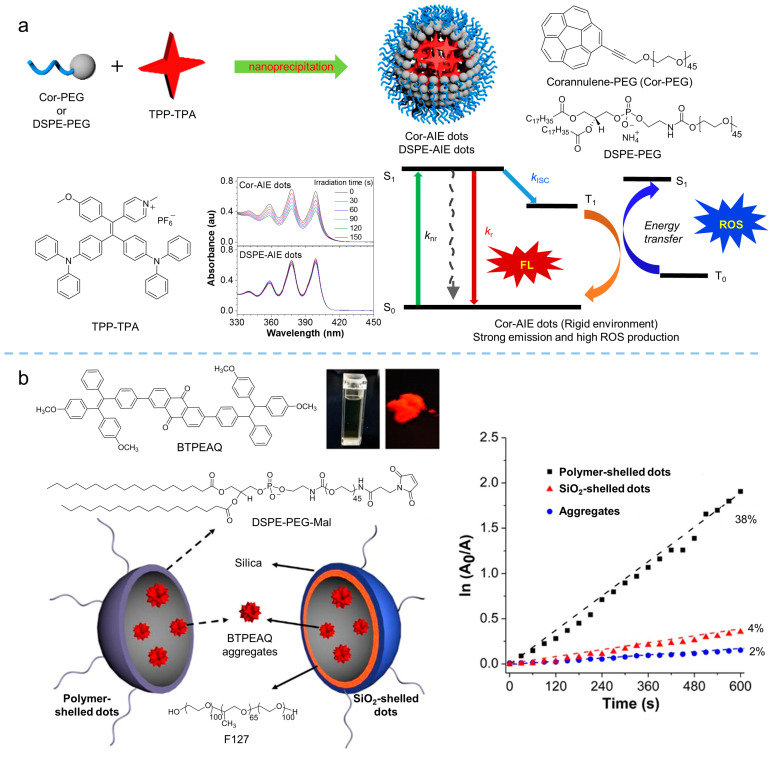
Aggregation microenvironment strategies to promote ROS production. (**a**) Schematic illustration of the construction of the Cor-AIE dots and DSPE-AIE dots with the photosensitizer of TPP-TPA. ROS generation from the flexible (DSPE-AIR points) and rigid (core points) aggregation environment, and the mechanism illustration of ROS manipulation. Reprinted with the permission of the authors of [73]. Copyright 2018, Wiley-VCH GmbH. (**b**) Molecular structure of BTPEAQ and schematic illustration of polymer and SiO_2_-shelled dots; degradation of ABDA by BTPEAQ along with increased irradiation time. Reprinted with the permission of the authors of [74]. Copyright 2016, American Chemical Society.

The aggregation microenvironment exhibits significant influence on the performance of photosensitizers, relying on the intermolecular interaction between photosensitizers or photosensitizer and guest molecules. By tuning the aggregation microenvironment of an AIE-active photosensitizer, ROS generation can be manipulated. In our previous work [73], we proposed a facile approach to manipulating the performance of photosensitizers via altering the aggregation microenvironment of photosensitizers, as shown in Figure 13a. Corannulene is a conjugated molecule with large steric hindrance and a more rigid structure than that of the alkyl chain. By synthesizing the Cor-PEG comprising corannulene in the end of the PEG chain and taking Cor-PEG as the polymer matrix to encapsulate the photosensitizer of TPP-TPA (*λ*_ex_ = 440 nm, *λ*_em_ = 680 nm) via nanoprecipitation, nanodots of the photosensitizer with a more rigid aggregation microenvironment in comparison with that of the DSPE-PEG encapsulated nanodots were obtained. The results demonstrated that rigidification of the aggregation microenvironment in Cor-PEG dots facilitated ROS production with a 5.4-fold enhancement compared to that of DSPE-PEG dots. Hence, this strategy, relying on aggregation microenvironment manipulation, provided a facile way to tailor ROS production without complicated chemical synthesis, exhibiting the potential for enhanced PDT. Similarly, Liu [74] found that the aggregation of BTPEAQ (*λ*_abs_ = 335 nm, *λ*_em_ = 650 nm) could not generate ROS in water, while ^1^O_2_ detected as BTPEAQ was encapsulated in the polymeric matrix with polymer or SiO_2_ shell. The different shells led to the different aggregation microenvironments, which can be used to tune the photosensitization of BTPEAQ aggregates. The results, as shown in Figure 13b, indicated that a higher ROS productivity could be achieved in the polymeric matrix encapsulated BTPEAQ than in the SiO_2_ matrix. Therefore, the manipulation of aggregation microenvironments is a simple but effective strategy for tuning the high performance of aggregate photosensitizers.

## 3. Advanced Development in PDT

Compared with traditional cancer treatment modalities, such as radiotherapy and chemotherapy, PDT exhibits the advantages of safety and non-invasiveness. Employing photosensitizers to generate ROS under a certain wavelength of light irradiation, PDT has been widely implemented in oncotherapy. However, the efficiency of PDT relies on the performance of the photosensitizers, and the development of the photosensitizers has dominated the improvement of PDT. In this section, we review the cutting-edge progress achieved by PDT based on the AIE-active photosensitizer, including NIR-absorbent PDT, activatable PDT, hypoxic PDT, and synergistic therapy.

### 3.1. Near Infrared Absorbent PDT

**Figure 14:**
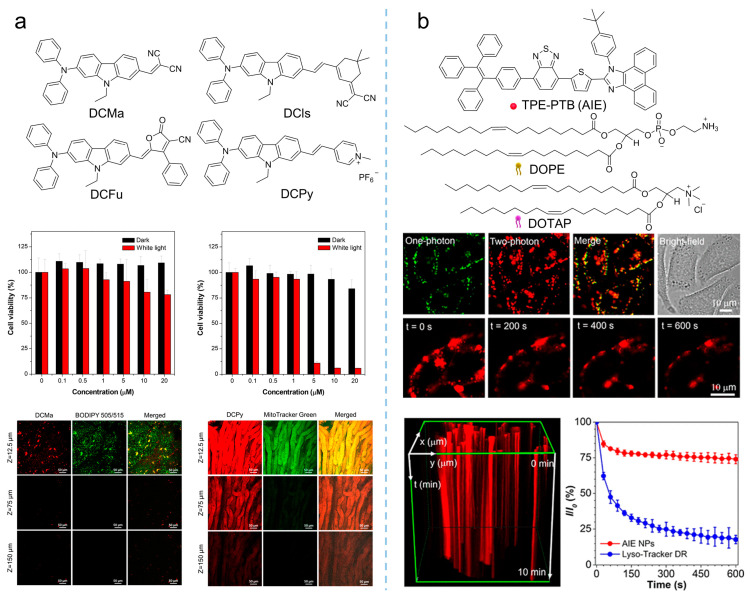
Multiphoton NIR absorption to enhance PDT theranostics. (**a**) Synthetic routes to DCMa, DCIs, DCFu, and DCPy and the in-vivo application of photosensitizers on the hepatic and nephric tissue of mice upon a 900 nm two-photon irradiation. Reprinted with the permission of the authors of [76]. Copyright 2018, American Chemical Society. (**b**) Molecular structure of TPE-PTB NPs, and cell imaging and photostability of AIE nanoparticles in A375 cells under continuous two-photon laser irradiation. Reprinted with the permission of the authors of [77]. Copyright 2020, American Chemical Society.

Compared to visible light, NIR light (700–1700 nm) demonstrated greater superiority based on reduced photodamage, lower scattering, and deeper light penetration [67,75]. Therefore, photosensitizers with both excitation and emission in the NIR regions are ideal candidates for PDT, and more and more studies were conducted to develop new NIR-absorbent photosensitizers. Tang et al. [76] prepared a series of molecules with far-red and NIR emission, good two-photon absorption, and efficient ^1^O_2_ generation by modifying the electron-donating group of diphenylamine through an electron-rich carbazole ring with different electron-withdrawing groups (malononitrile, isophorone, methylpyridinium salt, and 3-cyano-4-phenyl-2(5H)-furanone), as shown in Figure 14a. Notably, DCMa (*λ*_abs_ = 478 nm, *λ*_em_ = 665 nm), DCIs (*λ*_abs_ = 510 nm, *λ*_em_ = 709 nm), and DCFu (*λ*_abs_ = 538 nm, *λ*_em_ = 755 nm) exhibited the ability to specifically target lipid droplets (LDs), while the cationic lipophilic DCPy (*λ*_abs_ = 454 nm, *λ*_em_ = 698 nm) was endowed with excellent mitochondrion-specific targeting ability. Upon cellular imaging, all molecules exhibited good biocompatibility and high contrast, as well as higher photostability than that of commercial probes. The efficient ^1^O_2_ generation of these fluorophores (especially DCPy) under white light irradiation can be applied to effective PDT, demonstrating the potential of AIE-active photosensitizers as two-photon fluorescence-imaging agents for imaging-guided PDT. Similarly, as shown in Figure 14b, by encapsulating the AIE-active TPE-PTB (*λ*_ex_ = 488 nm, *λ*_em_ = 601 nm) within lipids, Tang et al. [77] prepared special nanoparticles with two-photon absorption, bright far-red emission, high quantum yield, and efficient ^1^O_2_ and •OH release. The obtained nanoparticles exhibited a maximum two-photon absorption cross-section (*δ*) of 560 GM at NIR region with a quantum yield of 23%, demonstrating high-resolution deep (up to 505 μm) tumor-imaging capability as they accumulated at the tumor. In addition, the AIE nanoparticles demonstrated excellent PDT efficiency in high ROS generation, indicating their great potential as powerful and safe therapeutic agents for oncotherapy. In addition to the photosensitizers mentioned in the works listed here, various NIR-absorbent photosensitizers have been developed, which have become the cutting-edge area for AIE-active photosensitizers [68].

### 3.2. Activatable PDT

**Figure 15:**
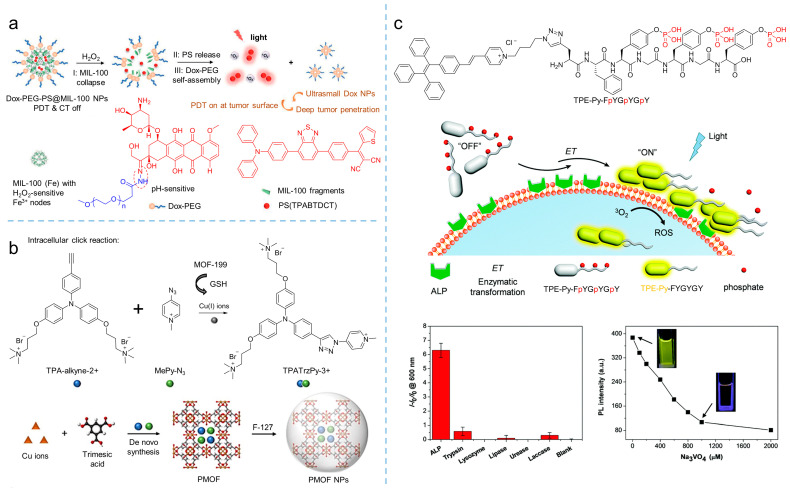
Activatable photosensitizers to enhance theranostics. (**a**) Schemes of MIL-100 collapse, PS release, and Dox-PEG self-assembly of Dox-PEG-PS@MIL-100 nanoparticles to tune PDT in H_2_O_2_. Reprinted with the permission of the authors of [81]. Copyright 2021, Wiley-VCH GmbH. (**b**) Scheme of the synthesis of TPATrzPy-3+ by two photochemically inert precursors under the catalysis of copper (I) ions generated from GSH-reduced-MOF-199. Reprinted with the permission of the authors of [82]. Copyright 2021, Wiley-VCH GmbH. (**c**) Schematic illustration of the self-assembly of TPE-Py-FpYGpYGpY under the catalysis of ALP, which significantly activates fluorescence and ROS generation. Reprinted with the permission of the authors of [83]. Copyright 2018, Royal Society of Chemistry.

Designing activatable photosensitizers is an effective strategy for overcoming the uncontrolled phototoxicity of photosensitizers as they are implemented in vivo, providing smart oncotherapy for clinical applications. Owing to the rapid proliferation and vigorous metabolism of cancer cells, the tumor microenvironment always contains overexpressed factors, such as H_2_O_2_, GSH, hydrogen ion, and some enzymes, making it possible to construct activatable PDT [78,79,80]. As shown in Figure 15a, Liu et al. [81] synthesized an AIE-active photosensitizer (*λ*_abs_ = 480 nm, *λ*_em_ = 690 nm), followed by loading it into iron (III) carboxylate-based MOF, MIL-100 to produce PS@MIL-100. After that, a pH-sensitive doxorubicin (Dox)-PEG matrix was synthesized by Dox and PEG via a hydrazone bond. Using the self-assembly of Dox-PEG, PS@MIL-100 was encapsulated to prepare Dox-PEG-PS@MIL-100 nanoparticles. As the Dox-PEG-PS@MIL nanoparticles reached the tumor site, intertumoral H_2_O_2_ disorganized the nanoparticles and released the loaded photosensitizer on the tumor surface, and PDT was eventually triggered in the tumor.

GSH is considered as a reducing agent to balance intracell ROS production, and it maintains a high concentration due to vigorous metabolism, which is usually used to conduct activatable PDT. By introducing a GSH-activated click reaction, Liu et al. [82] developed an MOF-assisted activatable photosensitization system for targeted PDT. In their work, the Cu(II)-based metal-organic framework, MOF-199, was used as the carrier to load the reactants TPA-alkyne-2+ and MePy-N_3_, and then encapsulated by F127 to obtain PMOF nanoparticles. The obtained nanoparticles were effectively enriched in the tumor and destroyed by GSH. As a result, the Cu(II) in the MOF was reduced into Cu(I), consequently releasing the reactants of TPA-alkyne-2+ and MePy-N_3_. Catalyzed by Cu(I), the click reaction was initiated and the photosensitizer of TPATrzPy-3+ was obtained. Additionally, under light irradiation, mitochondrion-targeted TPATrzPy-3+ (*λ*_ex_ = 400 nm, *λ*_em_ = 595 nm) with an efficient generation of ^1^O_2_ exhibited excellent PDT effectiveness.

Enzymes are another factor used for constructing activatable PDT. Recently, Ding et al. [83] synthesized the AIE-active photosensitizer of TPE-Py- FpYGpYGpY by modifying a short peptide with three tyrosine phosphates (pY). Due to the hydrophilic phosphotyrosine residue, the TPE-Py-FpYGpYGpY dissolved into water, resulting in weak fluorescence and negligible ROS generation. However, upon exposing TPE-Py-FpYGpYGpY in alkaline phosphatase (ALP) circumstances, an enzymatic cleavage of dephosphorylated precursors of TPE-Py-FpYGpYGpY was triggered, and the ALP-catalyzed products were self-assembled, resulting in the ROS-active photosensitizer (*λ*_ex_ = 405 nm, *λ*_em_ = 600 nm), which demonstrated the potential of enzymatic-activated PDT.

### 3.3. Hypoxic PDT

**Figure 16:**
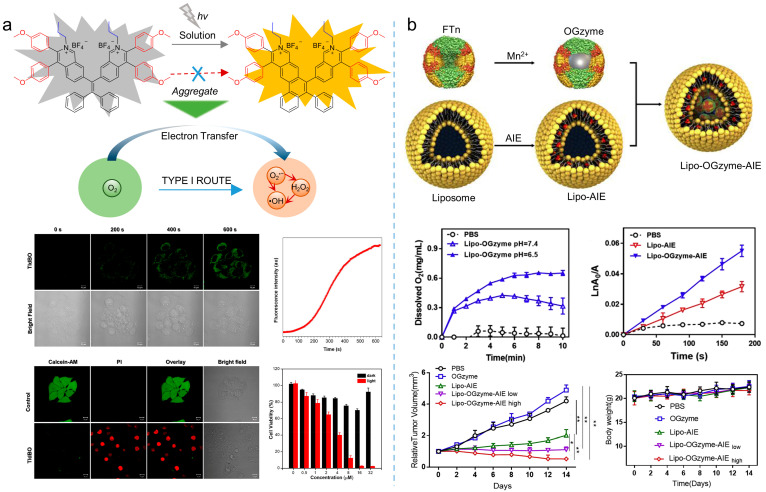
Type I ROS or self-oxygenated strategies to enhance theranostics. (**a**) Schematic illustration of producing type I ROS utilizing the electron transfer during the photoactivation process and theranostics effects of type I photosensitizers. Reprinted with the permission of the authors of [46]. Copyright 2021, American Chemical Society. (**b**) Schematic illustration of the preparation of nanoparticles, cascade reactions induced by the nanozymes and oxygen production, ROS production, and therapeutic effect of the nanozymes. Reprinted with the permission of the authors of [86]. Copyright 2020, Elsevier.

Oxygen concentration in tumor tissue is varied, depending on tumor progression, angiogenesis, metabolism, and metastasis. However, hypoxia of the microenvironment is a characteristic feature of solid tumors. There is increasing evidence that PDT efficiency that relies on traditional photosensitizers is limited, due to the oxygen dependence of those photosensitizers [84]. Therefore, the development of therapeutic strategies to alleviate tumor hypoxia, including catalyzing intracellular substrates to produce oxygen and promoting ROS production through type I mechanism, have become the potential solutions. Qi et al. [85] synthesized the electron-rich MeOTPPM (*λ*_abs_ = 452 nm, *λ*_em_ = 667 nm) with a donor-acceptor structure, and proposed the concept of “more ICT effect in electron-rich anion-π^+^ AIEgens, more effectively generate free radical ROS”. Through their strategy, the synthesized MeOTPPM facilely generated type I ROS, exhibiting O_2_ independence and an excellent PDT effect in a hypoxic microenvironment. In addition, MeOTPPM could specifically target mitochondria without the help of any additional targeted ligands, and its selective accumulation within cancer cells made it more PDT-efficient against cancer cells. Furthermore, Tang et al. [46] synthesized an electron-rich isoquinoline organic salt derivative, TIdBO, with excellent photoactivation properties, as shown in Figure 16a. From their study, the production of type I ROS from TIdBO could be significantly enhanced, due to the electron transfer during the photocyclization reaction. It is worth noting that the fluorescence intensity was enhanced after the ROS were produced and the apoptosis of cancer cells was subsequently triggered, realizing the self-monitoring of the PDT process.

Except for producing type I ROS, strategies based on oxygen self-sufficient nanoplatform could be an alternative for overcoming hypoxic limitations. For example, as shown in Figure 16b, Huang et al. [86] prepared a hypoxia-tropic nanozyme as an oxygen generator (OGzyme) by the biomimetic synthesis of MnO_2_ nanoparticles inside the hollow cavity of ferritin nanocages (FTn). After that, the obtained OGzyme and the AIE-active photosensitizer were encapsulated into phospholipid bilayers. As the OGzymes aggregated at the tumors’ tissue, they provided sufficient oxygen for the photosensitization of an AIE-active photosensitizer by utilizing the H_2_O_2_ response of MnO_2_. Hence, this system worked in hypoxic tumor tissue, and exhibited a strong PDT effect. Similarly, Liu et al. [87] developed carrier-free hybrid nanospheres comprising an AIE-active photosensitizer, iron ions, and a Bcl-2 inhibitor for hypoxic tumor PDT. Based on this strategy, the intracellular O_2_ concentration was increased via a Fe^3+^-derived Fenton reaction. Moreover, by inhibiting the production of Bcl-2 protein, intracellular ROS of the tumor was increased, and the PDT efficiency was improved synergistically.

### 3.4. Synergistic Therapy

**Figure 17:**
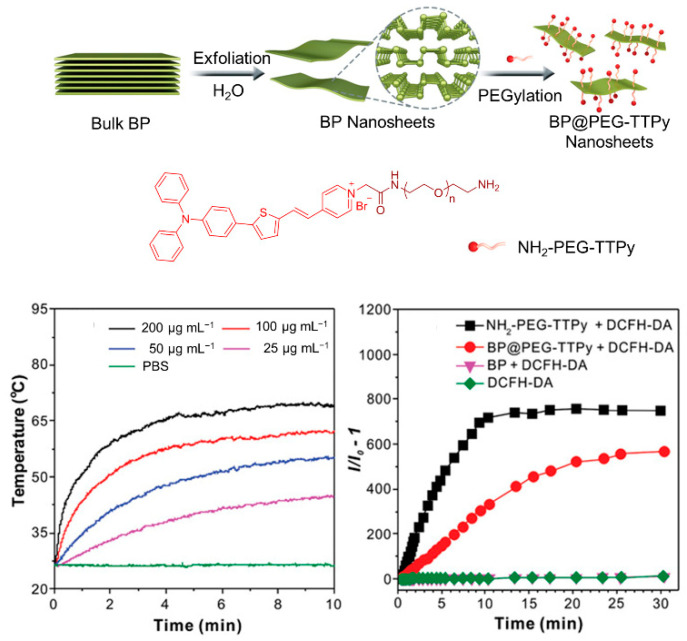
Synergistic therapy combining PDT. Schematic illustration of the preparation processes of BP@PEG-TTPy nanosheets and PDT/PTT effects of nanosheets. Reprinted with the permission of authors of [88]. Copyright 2020, Wiley-VCH GmbH.

The therapeutic efficiency of a single modality of PDT is often limited. It is crucial to develop a multifunctional treatment system for the synergistic therapy of tumors. Currently, the commonly used strategy for constructing multimodal optical therapeutic systems is a combination of multiple components in a single nanoplatform, such as synergistic photothermal therapy (PTT)/PDT and synergistic chemotherapy/PDT. As shown in Figure 17, by combining the positively charged and hydrophilic AIE-active photosensitizer (NH_2_-PEG-TTPy) with the negatively charged surface black phosphorus nanosheets (BP nanosheets) via electrostatic interactions, Tang et al. [88] developed the nanomaterial of BP@PEG-TTPy (*λ*_ex_ = 488 nm, *λ*_em_ = 672 nm), which not only exhibited excellent stability, but also simultaneously integrated the advantages of the two components, including bright NIR fluorescence, efficient ROS generation, and the PTT effect. As a result, BP@PEG-TTPy achieves efficient imaging-guided PDT-PTT synergistic therapy under irradiation both in vitro and in vivo, demonstrating the significant improvement of synergistic therapy compared to that of single PDT.

**Figure 18:**
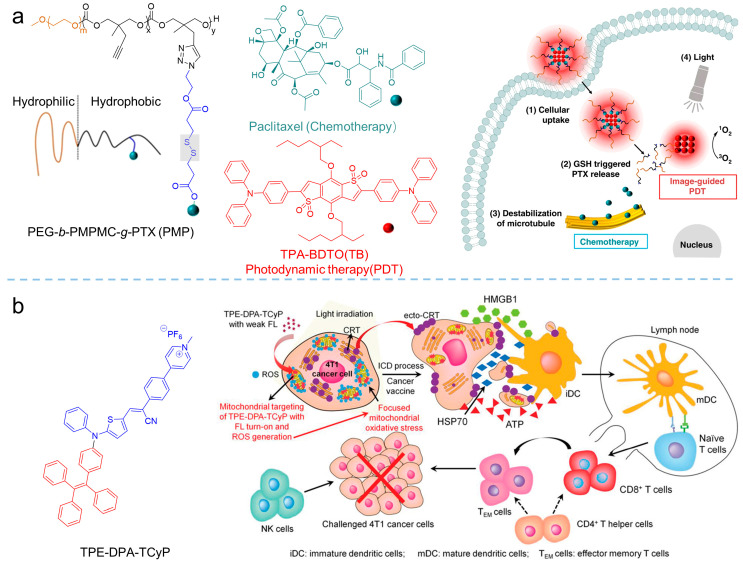
Synergistic therapy combining PDT. (**a**) Structure of the PEG-*b*-PMPMC-*g*-PTX (PMP) and TB@PMP (+), which was employed for combinational PDT/chemotherapy. Reprinted with the permission of the authors of [89]. Copyright 2018, by the author(s). Published by Springer Nature. (**b**) Schematic illustration of TPE-DPA-TCyP as an effective ICD inducer for antitumor immunity. Reprinted with the permission of the authors of [94]. Copyright 2019, Wiley-VCH GmbH.

Synergistic chemotherapy/PDT was also implemented in oncotherapy. Xia et al. [89] developed a reduction-sensitive co-delivery micelles TB@PMP for combinational therapy, where the chemotherapeutic drug, paclitaxel, was modified to the amphiphilic polymer through disulfide bonds, forming the reduction-sensitive polymer prodrug PMP, as shown in Figure 18a. The amphiphilic polymer prodrug PMP self-assembled into micelles in aqueous solution, and then encapsulated the AIE-active photosensitizer TPA-BDTO through a hydrophobic interaction. After that, TB@PMP (*λ*_ex_ = 530 nm, *λ*_em_ = 684 nm) micelles were passively enriched in the tumor site via the EPR effect. As the TB@PMP micelles were taken up by tumor cells, the disulfide bonds in PMP were cleaved, due to the high concentration of GSH in the tumor cells, resulting in the release of the paclitaxel. At the same time, TPA-BDTO produced cytotoxic ROS under light irradiation. As a result, when the ROS and the paclitaxel were combined, the balance between the microtubule aggregation and deaggregation was disrupted by the paclitaxel, which led to replication failure and, ultimately, the cancer cell apoptosis. Moreover, Singh et al. [90] prepared one-component organic nanoparticles for chemo-photodynamic therapy by conjugating a TPE moiety with a *p*-hydroxy phenacyl-chlorambucil conjugate. When exposed to visible light, the obtained nanoparticles simultaneously produced ^1^O_2_ and released the anticancer drug chlorambucil. The cytotoxicity assay showed that the anticancer activity of the obtained nanoparticles was significantly enhanced by the synergistic combination therapy.

A combination of PDT and immunotherapy has been emerging as a new strategy for cancer treatment [91]. In the processes of combined PDT and immunotherapy, calreticulin (CRT) would translocate to the cell membrane from the endoplasmic reticulum (ER) upon the stimuli of ROS, facilitated dendritic cell (DC) recruitment, recognition, and antigen presentation, to strengthen the host’s immune response [92,93]. Based on that, Ding et al. [94] synthesized the AIE-active photosensitizer of TPE-DPA-TCyP (*λ*_abs_ = 504 nm, *λ*_em_ = 697 nm) with mitochondrial targeting. Employing the ROS generation of the TPE-DPA-TCyP, the mitochondrial oxidative stress rose and resulted in immunogenic cell death (ICD). In their work, the effective in vivo ICD immunogenicity of TPE-DPA-TCyP was demonstrated using a prophylactic tumor vaccination model, revealing that intracellular oxidative stress in mitochondria could also cause ICD, rather than the commonly accepted ICD that originated from ER. The comprehensive mechanism of TPE-DPA-TCyP in ICD processes was verified by immune cell analyses, which provided a highly effective strategy for evoking abundant and large-scale ICD. Additional related works have been reviewed by Li [26] and Ding [95].

## 4. Summary

As a noninvasive treatment modality, PDT has been widely used in tumor and antibacterial therapies for decades; some of these applications have been approved by the Food and Drug Administration for clinical treatment. Photosensitizers are the most important part of the PDT process. However, the limitations of traditional photosensitizers are obvious, including ACQ effects in the aggregated state, short absorption wavelength, poorly produced ROS, and oxygen dependence. In contrast to traditional photosensitizers, AIE-active photosensitizers overcome some of these difficulties, endowing the photosensitizer with greater flexibility and allowing for further modification. Many AIE-active photosensitizers have been developed for clinical treatment, with features such as bright emission, effective ROS generation, a large Stokes shift, and far red/NIR fluorescence. In this review, we considered the development of AIE-active photosensitizers, involving the scope of the ROS generation mechanism, manipulation strategies for photosensitization, and advanced developments in PDT, which provided abundant information to guide molecular design and applications in the future. However, in perspective, there are still huge challenges with photosensitizers as they implemented in clinical treatments. The main focus is on advanced photosensitizers with excellent biocompatibility, deep penetration, and effective ROS generation. Additionally, the development of smart photosensitizers based on a facile way of manipulating ROS production, as well as the development of different types of ROS, will enhance PDT.
